# Editorial: The impact of non-communicable diseases on maternal health and pregnancy outcomes

**DOI:** 10.3389/fgwh.2026.1975383

**Published:** 2026-10-16

**Authors:** Karin Windsperger, Johanna Alexopoulos, Nadja Taumberger, Christian Göbl

**Affiliations:** 1 Department of Obstetrics and Gynecology, Medical University of Vienna, Vienna, Austria; 2 Department of Psychoanalysis and Psychotherapy, Medical University of Vienna, Vienna, Austria; 3 Department of Obstetrics and Gynecology, Barmherzige Brüder Hospital Graz, Graz, Austria

**Keywords:** diabetes mellitus, intergeneration, maternal health, non-communicable disease (NCDs), pregnancy outcome

The world is facing a global health crisis that remains far from under control. Noncommunicable diseases (NCDs) account for approximately 18 million deaths among women of reproductive age and an estimated 30.3 million disability-adjusted life years (DALYs) (1, 2). Their growing prevalence is also reshaping maternal health, with NCDs increasingly contributing to maternal morbidity and mortality (2). According to current WHO estimates, hemorrhage remains the leading cause of maternal death, followed by indirect causes, including NCDs (2). These figures underscore the growing importance of NCDs in modern obstetric care.

Yet, the approach to NCDs in pregnancy remains poorly articulated in maternal health literature. NCDs are generally defined as chronic conditions with variable temporal patterns, but no standardized definition has been established (3). The term encompasses a broad spectrum of conditions, including cardiovascular disease, obesity, diabetes, thyroid disease, malignancies, multiple sclerosis, and mental health disorders, many of which have important implications for maternal and fetal health (1). The physiological demands of pregnancy may modify the course of these conditions, while pre-existing disease may, in turn, increase the risk of adverse pregnancy outcomes. Importantly, the implications of NCDs extend beyond pregnancy itself, with potential consequences for maternal health across the life course and for the development and long-term health of the child.

The far-reaching consequences of NCDs underscore the need for an integrated approach to their prevention, recognition, and management across the reproductive life course. This Research Topic brings together a broad spectrum of current evidence and perspectives, highlighting the multiple pathways through which NCDs intersect with pregnancy and maternal and fetal health.

NCDs arise from a complex interplay of genetic susceptibility, lifestyle and individual behaviors, social and economic circumstances, environmental exposures, and global health determinants (1, 3). The contribution by Yan et al. highlights the importance of environmental determinants, investigating the impact of exposure to fine particulate matter (PM2.5) on maternal health in a large cohort study. Their findings add to growing evidence that air pollution may affect multiple organ systems, including the cardiovascular, respiratory, nervous, immune, and renal systems. In particular, the authors demonstrated an association between PM2.5 exposure and alterations in serum creatinine, suggesting a potential impact on renal function. These findings reinforce the concept that the burden of NCDs cannot be understood solely through individual-level risk factors, but must also be considered within the broader context of environmental and global determinants of health.

NCDs may influence pregnancy at several levels, from the development of pregnancy complications, such as preeclampsia and gestational diabetes mellitus (GDM), to adverse maternal and fetal outcomes. Pre-pregnancy obesity and metabolic dysfunction, for example, are closely associated with an increased risk of GDM. Zhao et al. provide further insight into early metabolic risk stratification in their large prospective cohort study, demonstrating that the triglyceride-glucose (TyG) index measured in the first trimester was independently associated with the subsequent development of GDM. Such findings highlight how NCDs can complicate pregnancy, making the early identification of women at increased risk a clinical priority. Conversely, pregnancy itself may exacerbate underlying chronic conditions; however, evidence on this bidirectional relationship remains limited and appears to vary across different disease entities.

The impact of maternal NCDs, however, may extend beyond maternal health and pregnancy complications to the developing fetus. In their narrative review, Sui et al. examine the relationship between maternal circulatory, endocrine and metabolic, infectious, and autoimmune diseases and the development of cleft lip and/or palate (CLP) in newborns. CLP are among the more common congenital anomalies, although their prevalence varies considerably across populations. By bringing together evidence on diverse maternal disease pathways, the review illustrates the complex interplay between maternal health and fetal development. Importantly, effects of NCDs (e.g., obesity) may extend beyond fetal development, with potential long-term health consequences for the offspring. Pregnancy therefore represents an important window during which maternal health may shape health outcomes across generations.

NCDs can also influence the course and outcome of delivery itself. In a retrospective cohort study, Zhuang et al. investigating hypothyroidism and severe postpartum hemorrhage (SPPH) found that overt hypothyroidism was significantly associated with an increased risk of SPPH, whereas no significant association was observed for subclinical hypothyroidism. Age and a history of radiation exposure appeared to modify this relationship, with a stronger association observed among women younger than 35 years. These findings underscore the importance of considering chronic maternal conditions not only in antenatal risk assessment but also in the planning and management of childbirth and the postpartum period.

While common NCDs account for a substantial proportion of the disease burden in pregnancy, less prevalent chronic conditions should not be overlooked. Deng et al. draw attention to a particularly rare and challenging group of disorders—pheochromocytomas and paragangliomas (PPGLs)—through a bibliometric analysis of the existing literature. Their analysis highlights the diagnostic and therapeutic challenges associated with PPGL during pregnancy, as well as the continued focus on pregnancy-related complications and clinical management. The rarity and complexity of these conditions make multidisciplinary collaboration particularly important and emphasize the need for clear clinical management pathways to ensure timely diagnosis and coordinated care for affected women.

Taken together, the contributions to this Research Topic illustrate the remarkable breadth of the intersection between NCDs and pregnancy—from the prevention and identification of risk factors to their effects on pregnancy and delivery and their potential consequences for maternal and fetal health (Figure 1). Moving the field forward will require not only improved understanding of these complex interactions, but also the development of multidisciplinary clinical pathways and evidence-based guidelines that integrate obstetric care with expertise from relevant medical specialties. Such approaches are essential to ensure that women with chronic conditions receive timely, coordinated, and individualized care before conception, throughout pregnancy, and beyond.

**Figure 1:**
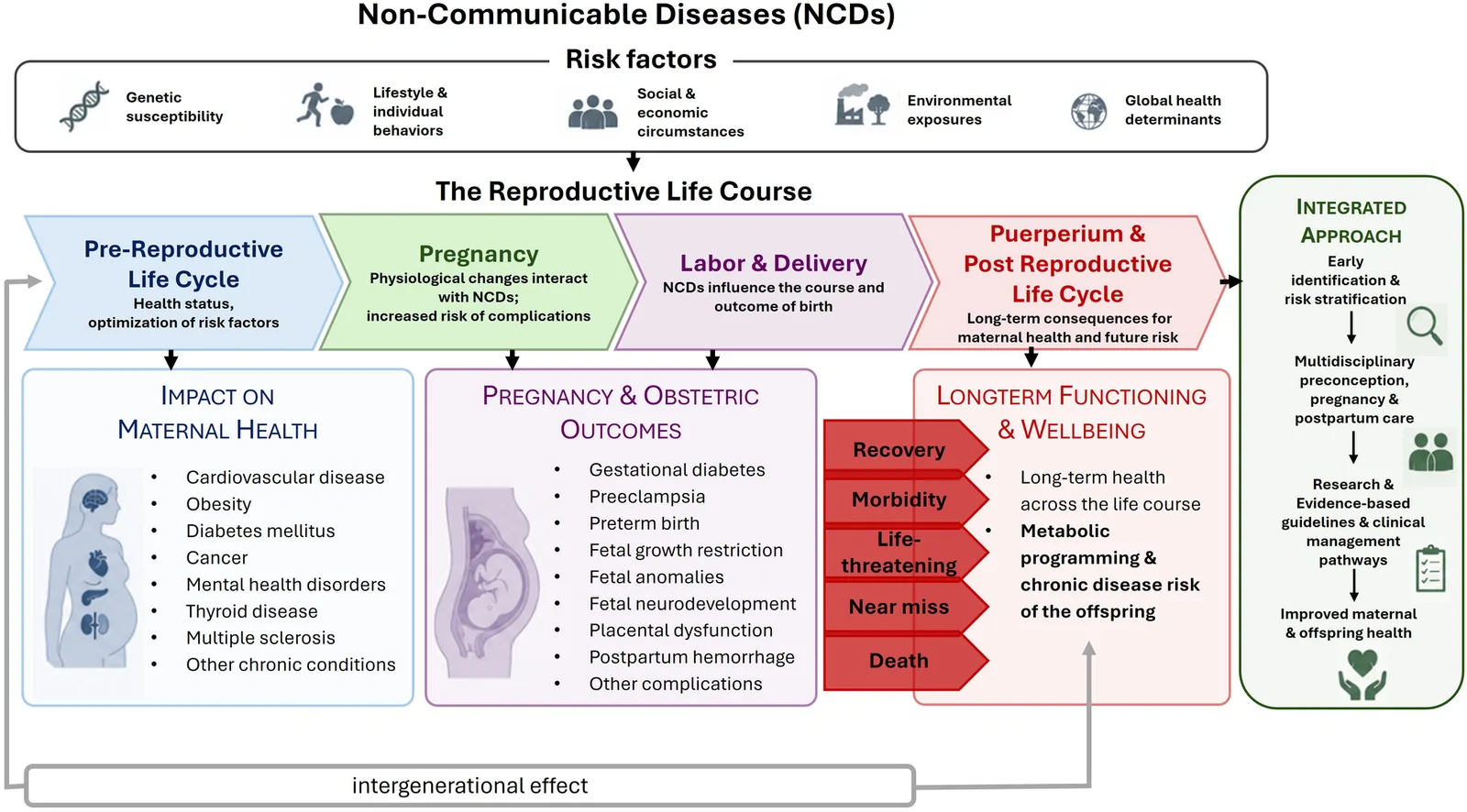
Non-communicable diseases (NCDs) across the reproductive life course, from preconception through pregnancy and beyond, and their implications for maternal and fetal health. An integrated, multidisciplinary approach is essential to prevent complications and improve outcomes for mothers and their children.

## References

[1] Barnes SB Ramanarayanan D Amin N. The Unseen Side of Pregnancy: Non-communicable diseases and Maternal Health. Washington, DC: Maternal Health Initiative, Wilson Center (2020). Available online at: https://www.wilsoncenter.org/publication/unseen-side-pregnancy-non-communicable-diseases-and-maternal-health (cited August 19, 2026).

[2] Cresswell JA Alexander M Chong MYC Link HM Pejchinovska M Gazeley U. Global and regional causes of maternal deaths 2009-20: a WHO systematic analysis. Lancet Glob Health. (2025) 13(4):e626–e34. 10.1016/S2214-109X(24)00560-6 40064189 PMC11946934

[3] Firoz T Pineles B Navrange N Grimshaw A Oladapo O Chou D. Non-communicable diseases and maternal health: a scoping review. BMC Pregnancy Childbirth. (2022) 22(1):787. 10.1186/s12884-022-05047-6 36273124 PMC9587654

